# Where are we in family-centered intervention? Parental experiences of DHH children

**DOI:** 10.1093/jdsade/enaf081

**Published:** 2025-12-31

**Authors:** Murat Doğan, Mahire Kılıç, Ramazan Bekar

**Affiliations:** Faculty of Education, Department of Special Education, Anadolu University, Eskişehir, Türkiye; Faculty of Education, Department of Special Education, Anadolu University, Eskişehir, Türkiye; Faculty of Education, Department of Special Education, Muğla Sıtkı Koçman University, Muğla, Türkiye

**Keywords:** deaf and hard-of-hearing children, early intervention, family-centered early education, parent education

## Abstract

There are global initiatives aimed at improving the quality of Family-Centered Early Education (FCEE) for deaf and hard-of-hearing (DHH) children and their parents. This study aims to examine the FCEE practices provided to parents of DHH children aged 0–3 in Türkiye based on parental experiences. Employing a sequential explanatory mixed methods design, data were collected from 268 parents in the quantitative phase and 10 in the qualitative phase through the FCEE-DHH Survey, semi-structured interviews, and information form. Descriptive analyses were conducted, with qualitative findings elaborating on the quantitative results. Findings were categorized into four dimensions: information, guidance, intervention, and assessment. Results indicated that parents primarily relied on internet sources and peer networks to address informational needs. For guidance, limited access to psycho-social support and a lack of attention to parents’ emotional needs were reported. Intervention practices were predominantly child-centered with minimal parental involvement. The absence of a systematic assessment led to unrealistic expectations regarding children’s development. These results underscore the need for comprehensive, interdisciplinary FCEE programs in Türkiye supported by legal frameworks and integrated service policies.

Hearing loss is observed in 1 to 3 out of every 1,000 newborns (World Health Organization [WHO], 2018). Congenital hearing loss limits access to auditory input and linguistic cues, which makes spoken language acquisition more difficult (Dunst & Espe-Sherwindt, 2017; Yoshinaga-Itano et al., 2018). In addition to language development, hearing loss may lead to delays in communication skills, social–emotional, and cognitive development (Tomblin et al., 2015). However, these delays can be mitigated when early intervention services are accessed in a timely manner (Oliveira et al., 2015; Yoshinaga-Itano et al., 2020).

For deaf and hard-of-hearing (DHH) children, early intervention based on an auditory-verbal approach typically begins at diagnosis and includes medical/audiological intervention and family-centered early education (FCEE; Joint Committee on Infant Hearing [JCIH], 2019). This study focuses on the FCEE component of early intervention and adopts the FCEE framework in line with international literature. Within FCEE, early education services are provided to families through guidance, coaching, and counseling. In Türkiye, such services targeting children aged 0–3 are officially called parent education (Ministry of National Education [MoNE], 2018). In Western countries, such as Norway and the United States, efforts have been made to establish standards for early intervention for DHH children (Moeller et al., 2024a). Therefore, assessing the quality of early intervention services in Türkiye is essential for meeting these international standards.

## FCEE

For FCEE to be effective, it is recommended that hearing screening take place at birth, hearing loss be diagnosed by the third month, and early intervention begin no later than the sixth month (JCIH, 2019; Moeller et al., 2024a; Walker et al., 2022). In recent years, family-centered approaches have become central to early intervention programs, which emphasize the importance of active involvement from both the child and their social environment in the developmental process (Holzinger et al., 2020; Ingber & Dromi, 2010; Szarkowski et al., 2024).

In early intervention for DHH children, stakeholders from various countries have widely agreed that family-centered practices are the most effective approach (Moeller et al., 2024a; Moodie et al., 2024). The decisions made during an international consensus panel reflect a global effort to establish standards for FCEE. The accepted quality standards for early intervention include: accessible resources (Moeller et al., 2024a; Moodie et al., 2024), strong collaboration between parents and specialists, empowering parents as key decision-makers (Hopkins & Puhlman, 2025), connecting families with psycho-social support resources (Moeller et al., 2013; Moeller et al., 2024b; Moodie et al., 2024), providing ample opportunities for language development, educating parents about hearing technologies, promoting transdisciplinary teamwork among service providers (Szarkowski et al., 2024), and ensuring the competence of specialists delivering FCEE (Moeller et al., 2013). These standards emphasize the critical role of the family in achieving high-quality outcomes in early intervention.

The family is a crucial environmental factor in a child’s development. According to ecological theory, individuals within the microsystem, such as parents and siblings, influence the child both directly and reciprocally (Bronfenbrenner & Evans, 2000). From a sociocultural perspective, the quality of a child’s interactions with their parents and linguistic environment plays a critical role in language development (Vygotsky, 1978). In addition, during the critical period of language acquisition, the family’s early interactions, as the child’s first social environment, are particularly significant (Bruder & Dunst, 2005; Noll et al., 2021). On the other hand, family systems theory provides a framework for understanding the impact of a new member on family interactions and functioning, and how support provided to parents spreads to the entire family (Odom, 2016). To support parent–child interactions, early intervention should adopt a holistic approach that considers the entire family (Moeller et al., 2013). Given the family’s undeniable role and supporting research findings, mitigating the negative impacts of hearing loss appears achievable through FCEE (Holzinger et al., 2011; Moeller et al., 2013). FCEE practices support DHH children’s language development and parents’ communication behaviors (Costa et al., 2019; Szarkowski et al., 2024; Walker et al., 2022). Parents’ developing communication skills also positively influence their interactions with their children (Reichmuth et al., 2013; Sass-Lehrer et al., 2016; Zauche et al., 2016). Additionally, parents who participated in FCEE demonstrated positive attitudes (Stika et al., 2015).

A closer look at the research and established standards reveals that FCEE services are typically organized into four main areas: information, guidance, intervention, and assessment.

### Information

FCEE is recommended to begin no later than the sixth month following the birth of a DHH child (JCIH, 2019). During this period, parents are not only required to make important decisions but also must cope with the emotional and practical challenges associated with their child’s diagnosis (Athalye et al., 2015). Notably, 90%–95% of DHH children are born to hearing parents (Dirks et al., 2019), meaning most families are encountering hearing loss for the first time. This unfamiliar situation often triggers a range of emotional responses, such as sadness, anxiety, and guilt, and creates an urgent need for accurate information. It is essential to address their informational needs early in the process to help parents adapt to this new reality and make informed decisions for their child (Athalye et al., 2015; Cankuvvet & Doğan, 2025; Young et al., 2006). Providing clear, timely, and accurate information about the nature and impact of hearing loss, language development, hearing technologies, and available early intervention options is strongly emphasized in the literature (Clark, 2007; van der Spuy & Pottas, 2008). In this context, informed guidance serves as a foundation for empowering parents to make sound, confident decisions regarding their child’s care (Bruder & Dunst, 2005; Decker et al., 2012).

### Guidance

Due to the impact of hearing loss on the child, parents often require various forms of support during the early stages. These needs typically include audiological, medical, educational, and psycho-social support within the scope of early intervention (Cankuvvet & Doğan, 2025; Fitzpatrick et al., 2025; Kecman, 2022; Luterman, 2017). It is recommended that families receive guidance on how to access these resources based on their specific needs (Bekar et al., 2021; Moeller et al., 2013).

### Intervention

Specialists play a crucial role as role models by equipping parents with the skills needed to support their child’s language development through natural, everyday interactions (Clark, 2007; Costa et al., 2019). Parents should be encouraged to involve their children in routines, activities, and play that promote development in listening, language (Clark, 2007), as well as cognitive, social–emotional (Vygotsky, 1978), and early literacy skills (Cole & Flexer, 2020; Miller et al., 2008). These recommendations should be tailored to the unique needs and cultural context of both the child and the family. Additionally, goals should be clearly communicated and collaboratively established with the parents (Brown & Nott, 2005; Moodie et al., 2024; Nickbakht et al., 2021).

### Assessment

In the early stages, parents may have unrealistic expectations for age-appropriate progress in their child’s language development. When these expectations are not met, it can lead to frustration and inaction (Doğan, 2015; Luterman, 2017; Mauldin, 2019). Therefore, it is important to help parents understand the timeline and extent of their child’s developmental progress as part of the parent education process (Clark, 2007; Moeller et al., 2013). Specialists play a key role in helping parents recognize their child’s achievements, assess the situation, and become attentive observers of their child’s development. This active involvement and motivation are essential for sustaining the family’s engagement in the intervention process (Dunst & Espe-Sherwindt, 2017).

## FCEE practices in Türkiye

FCEE practices are conducted in a variety of institutions, including university-affiliated centers (e.g., Applied Research Center for Hearing Impaired Children [namely, İÇEM]), clinics, independent centers (e.g., MoNE and the Ministry of Family and Social Services), associations (e.g., AG Bell Academy), and early intervention centers (Estabrooks et al., 2016; Estrella-Castillo et al., 2020; Geers et al., 2019; Kendrick & Smith, 2017; Moeller et al., 2013). In Türkiye, medical diagnosis is carried out in parallel with international practices (JCIH, 2019) through the National Newborn Hearing Screening Program (NNHSP) (Kemaloğlu, 2015). Following early diagnosis, children with hearing loss undergo screening through the NNHSP in their first month and proceed to the diagnostic and device fitting stage.

Following medical diagnosis, children are referred to Guidance Research Centers (GRCs) to identify an educational environment that aligns with their individual characteristics. GRCs are widespread institutions located in every city that are affiliated with the MoNE and provide educational diagnosis, referral, and placement recommendations. Educational diagnosis is performed at GRCs by specialists in special education and psychological counseling. Along with educational diagnosis, the child and family are directed to the most appropriate educational environment (Yılmaz & Doğan, 2023).

Special Education and Rehabilitation Centers (SERCs) stand out among the institutions where DHH children and their families receive education during the 0–3 age period (Ertürk Mustul et al., 2025). SERCs are free education centers supported by MoNE that provide extracurricular educational support. Educational support is provided in the areas of listening, language, social communication, learning, reading and writing, early mathematics, and mathematics modules (MoNE, 2024). DHH children and their families receive 8 hours of education per month at SERCs between the ages of 0–3 and increasing to 12 hours per month after the age of 3 (MoNE, 2018). In SERCs, parent education is delivered by teachers of the deaf, special education teachers, classroom teachers, preschool teachers, child development specialists, and audiologists (MoNE, 2018, 2024). Consequently, FCEE practices are commonly carried out in SERCs.

Telepractice is a form of FCEE that allows real-time communication between families, children, and specialists, helping to overcome barriers to educational access in rural areas (American Speech-Language-Hearing Association, 2019; Gerlach et al., 2023; McCarthy et al., 2020). In Türkiye, telepractice has been more commonly applied in speech and language therapy (Cangi & Toğram, 2020) and was first implemented for DHH children during the COVID-19 pandemic in university-affiliated institutions (e.g., İÇEM). However, a study highlighted limitations including low child engagement and insufficiently guided parent applications (Tutuk et al., 2025). Despite its potential, challenges such as lack of regulatory support (MoNE, 2018) and underdeveloped program hinder widespread adoption of telepractice. Further research and systematic implementation are necessary for telepractice to be accepted as a viable alternative to face-to-face education (Behl et al., 2017). Overall, FCEE practices in Türkiye vary considerably depending on institution and mode of delivery.

## Rationale

Globally, initiatives have been established to ensure consistency in early intervention practices for DHH children and to set standards for service delivery (JCIH, 2019; Moeller et al., 2013; Moodie et al., 2024). Although NNHSP is being successfully implemented in Türkiye, family needs remain prominent across the information, guidance, intervention, and assessment dimensions of FCEE practices.

Research conducted at application centers and SERCs reveal discrepancies between theory and practice due to the lack of systematic parent education programs. This difference is particularly evident in the education dimension, as families face difficulties in accessing quality education, qualified specialists, and SERCs (Baş et al., 2019; Bekar et al., 2021; Kılıç, 2020). While studies conducted in Türkiye examined aspects such as the FCEE guidance process (Baş et al., 2019), family needs in the information and education process (Bekar et al., 2021; Kılıç, 2020), the structure of SERCs (Atmaca & Uzuner, 2020), and the qualifications of education specialists (Ertürk Mustul et al., 2025) no research has yet captured the nationwide implementation of all FCEE dimensions from the perspective of families. The main rationale for this research was therefore to obtain an updated picture of FCEE practices in Türkiye alignment with international standards.

## Purpose

Understanding the extent to which international FCEE principles (Moeller et al., 2013; Moodie et al., 2024) are implemented is expected to enhance comprehension of FCEE practices. The research findings of this study are anticipated to serve as a foundational step toward creating systematic, accessible, and high-quality parent education programs in Türkiye that align with global standards. This study aims to examine the FCEE practices offered to parents of DHH children aged 0–3 in Türkiye regarding the experiences of the parents. The research questions are as follows:

1) To what extent is the content of FCEE (information, guidance, intervention, assessment) provided to DHH children and their parents?
2) What are parents’ perspectives on the FCEE practices they have received?
3) What metainferences can be drawn based on the level of FCEE content delivery and the views of parents regarding FCEE?

## Methods

### Design

**Figure 1:**
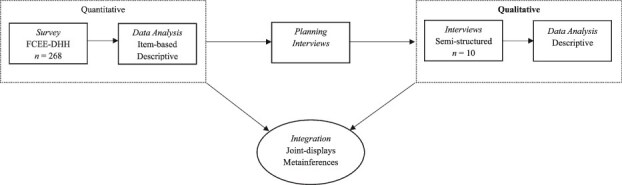
The research flow.

A two-phase research process was conducted to investigate the FCEE practices provided to DHH children in the 0–3 age range and their parents. The research flow is presented in Figure 1.

As shown in Figure 1, in the first stage, the FCEE-DHH Survey (FCEE-DHH) was administered to assess the extent to which FCEE was provided to parents across the dimensions of information, guidance, intervention, and assessment. Semi-structured interview questions were developed based on the survey data. In the second stage, semi-structured interviews were conducted with parents to explore their experiences, identify the underlying causes of the issues highlighted in the survey, and gather suggestions for improvement. In the final stage, quantitative and qualitative findings were integrated to draw comprehensive conclusions.

Since the quantitative findings were interpreted through qualitative analysis, the study follows a sequential explanatory design in mixed-methods research (Creswell & Plano Clark, 2023). Combining both quantitative and qualitative approaches allows for the simultaneous examination of multiple research questions, which leads to stronger and more comprehensive conclusions (Teddlie & Tashakkori, 2012). In addition, the use of multiple methods together provides triangulation, which allows data to support each other or, in cases of discrepancy, to clarify the reasons for divergence. Thus, completion, development, and expansion serve as methodological justifications for this study (Greene, 2007; Leko et al., 2023; Younas et al., 2023). In this study, the findings corresponding to the research questions were examined together, with particular emphasis on explaining discrepant results, and metainferences were drawn from the findings.

### Participants

In mixed-methods research, probability sampling strategies are typically used for quantitative data and purposive sampling strategies are employed for qualitative data (Teddlie & Tashakkori, 2012). Since probability sampling can limit access to the population within the relevant age range, the sample representativeness was prioritized, and key informants were utilized to reach participants across all regions of Türkiye.

Inclusion criteria: (a) children must be between the ages of 0 and 6, (b) parents must have participated in FCEE when their children were between the ages of 0 and 3, and (c) parents and children had received FCEE through an auditory-verbal approach. Although the primary focus was on children aged 0–3 years, the age range was extended to 0–6 years to include both parents currently enrolled in FCEE and those who had recently completed the process, which ensures that their experiences were still recent. For parents of children aged 4–6, the following instruction was included to the survey: “This section includes statements about the quality of the education you and your child participated in when your child was 0-3 years old.” The descriptive statistics of the survey participants are presented in Table 1.

**Table 1:** Descriptive statistics of survey participants (*n* = 268).

Categorical variables	*n*	*%*
Parent’s relation to the child		
Mother	214	79.9
Father	44	16.4
Other (grandparents, siblings)	10	3.7
Parents’ education		
Elementary education	84	31.3
Secondary education	96	35.8
Higher education	88	32.8
Gender of the child		
Female	132	49.3
Male	136	50.7
Hearing technology		
Not used	7	2.6
Hearing aid	82	30.6
Cochlear implant	160	59.7
Hearing aid + cochlear implant	19	7.1
Communication mode		
Oral language	196	73.1
Oral + sign language	72	26.9
Additional disabilities		
No	167	62.3
Yes	101	37.7
Family members with hearing loss		
None	178	66.4
Parent(s)	7	2.6
Sibling(s)	37	13.8
Relative	46	17.2
Educational institution (0–3 years)		
SERC	183	68.3
Clinic (university or private)	10	3.7
SERC + clinic	75	28.0
Early intervention specialist (0–3 years)		
Hearing-speech specialist	51	19.6
Non-hearing-speech specialist	41	15.8
Combination	168	64.6
Frequency of education (0–3 years)		
3 or below per month	84	31.3
4 or more per month	184	68.7
**Continuous variables**	***M***	***SD***
Age of the child (in years)	5.27	1.95
Child’s age at diagnosis (in months)	7.62	8.49

*Note.* SERC = special education and rehabilitation center.

As shown in Table 1, the majority of the 268 parents who participated in the survey were mothers. The distribution of parents’ educational levels was relatively balanced. Almost all children (97.4%) used hearing technology, and most parents and children received education from SERCs. Semi-structured interview data were collected approximately one month after the survey was conducted.

The interview participants consisted of 10 volunteer parents who had different experiences with FCEE in terms of their participation in the survey, closeness to their children (eight mothers and two fathers), educational status, age of their children, level of satisfaction with FCEE, and institutions they attended. All participants listed in Table 2 are hearing parents who use oral communication.

**Table 2:** Characteristics of semi-structured interview participants.

Participant	Education status	Child’s age	Hearing technology	Early intervention specialist	Educational institution	Frequency of ed. (per month)
(M1) Mother	Secondary	6	Cochlear implant	THI, PT, SLP, psychologist	SERC, kindergarten	≤ 3
(M2) Mother	Higher	5	Cochlear implant	CDS, audiologist	SERC	≤ 3
(F1) Father	Higher	6	Hearing aid	Audiologist, THI	SERC	≥ 4
(M3) Mother	Elementary	3	Cochlear implant	Audiologist	SERC	≥ 4
(M4) Mother	Higher	6	Cochlear implant	THI, PT	SERC, kindergarten	≥ 4
(M5) Mother	Higher	3	Hearing aid	SET, audiologist	SERC	≥ 4
(M6) Mother	Higher	5	Cochlear implant	SET, PT, audiologist	SERC, kindergarten	≥ 4
(F2) Father	Secondary	5	Hearing aid	CDS	SERC	≥ 4
(M7) Mother	Higher	6	Cochlear implant	THI, SET	SERC, İÇEM	≥ 4
(M8) Mother	Higher	3	Cochlear implant	THI, SLP, Audiologist	SERC, İÇEM	≥ 4

*Note.* CDS = child development specialist, İÇEM = applied research center for hearing impaired children, PT = preschool teacher, SERC = special education and rehabilitation center, SET = special education teacher, SLP = speech and language pathologist, THI = teacher of the hearing impaired.

As a strength of the sequential explanatory design, the researchers considered the positive and negative experiences identified through open-ended survey responses. They conducted semi-structured interviews with selected participants to gain deeper insights into these experiences. This approach allowed for the inclusion of diverse parental experiences related to FCEE, thereby enriching the interpretation and contextualization of the overall findings.

### Data collection tools/techniques

#### Participant information form

The Participant Information Form was used to collect demographic information about the parent, as well as demographic, educational, and audiological information about the child.

#### FCEE survey (FCEE-DHH)

The FCEE-DHH was developed to assess the characteristics of FCEE received by parents with children aged 0–3, and it consists of 40 items, including one open-ended item. The survey covers services expected to be provided at FCEE. Responses to the items are recorded as “Yes” or “No”, indicating whether the services were offered rather than evaluating their quality.

The development process followed established steps for instrument design, which include defining the research problem and objectives, conducting a comprehensive literature review, generating items, obtaining expert feedback, pilot testing, and making final revisions (Cohen et al., 2021; DeVellis & Thorpe, 2021). Drawing on the literature, the researchers identified four guiding categories for item development: information, guidance, intervention, and assessment. An initial pool of 89 items was generated, and seven meetings were held with three experts working with DHH children’s parents. In each session, the clarity, relevance, and coverage of the items were evaluated and revised accordingly. The revised draft was then reviewed by five additional experts not involved in the earlier stages to assess content validity and provide suggestions for further refinement. Following their feedback, a pre-pilot version of the FCEE-DHH was prepared. The pilot study involved 10 parents from three cities, five interviewed in person and five online. Based on pilot feedback, revisions were made to enhance clarity; for example, the phrase “cultural structure” was changed to “family characteristics” for improved comprehension. The final version of the FCEE-DHH includes 39 closed-ended items and one open-ended question and was deemed ready for data collection (Appendix A).

#### Semi-structured interviews

Semi-structured interviews are essential for gaining a deeper understanding of participants’ experiences related to the research topic (Tisdell et al., 2025). The interview questions were developed based on the study’s objectives, results from the quantitative analysis, responses to the open-ended question, a review of relevant literature, and the researchers’ own experiences. In formulating the questions, the researchers aimed to explore how the FCEE practices were implemented, how specialists delivered FCEE, and the positive and negative experiences parents encountered. The initial draft of the interview guide was reviewed by three experts with experience in qualitative research and FCEE. After incorporating their feedback, the interview questions were finalized (Appendix B).

A researcher’s diary consisting of 25 pages and 557 lines was kept before and after the semi-structured interview. The researcher’s diary served as a tool for documenting the researchers’ reflections, observations, plans, strategies, challenges, and successes throughout the research process (Glesne, 2016).

### Data collection procedure

Institutional and individual permissions were obtained prior to data collection. Ethical approval was granted by the Anadolu University Scientific Research and Publication Ethics Committee (Protocol No. 304133) and written informed consent forms were collected from all participants. Ethical approval was obtained from the University’s ethics committee, ensuring that the study met established research standards. Participants were fully informed about the study’s purpose, process, and their rights; and their identities were maintained confidential and anonymous throughout the research process.

During the quantitative data collection phase, key informants were contacted, and both printed and digital versions of the FCEE-DHH via Google Forms were distributed to parents of children attending SERCs in three cities. Additionally, the FCEE-DHH was shared in closed social media groups specifically for parents of DHH children to encourage wider participation. Using the Google Form helped reach participants who did not receive the printed survey and reduced the likelihood of missing data. Of the 320 surveys distributed, 282 were returned (88%). After excluding 14 surveys due to incomplete or inconsistent responses, the final sample included 268 participants for analysis. Following the quantitative analysis, participants for the semi-structured interviews were selected, and online interviews were conducted with 10 parents who volunteered. The interviews were conducted over a 26-day period, with the longest interview lasting 64 minutes and the shortest lasting 31 minutes.

### Data analysis

Quantitative data were subjected to descriptive analysis using the SPSS 25 software package. The FCEE-DHH was analyzed at the item level. In qualitative data analysis, descriptive analysis was conducted using the NVivo12pro software. The themes, code list, and related survey items are presented in Table 3.

**Table 3:** Common frame for quantitative and qualitative analysis.

Dimension/subdimension	Survey (item no.) and content	Qualitative codes
**Information**		
Nature of hearing Parent education process Role of social environment Setting realistic expectations	(1) Effects of hearing loss, (2) communication mode, (3) hearing technology, (31) sharing the content of education, (30) sharing the aims of education, (24) parental attitude, (4) family role in language development, (9) child’s place in the family, (8) role of social environment in language development, (7) approach to reactions to hearing loss, (25) expectations	Hearing loss, hearing technology, use of technology, educational approach, aims, planning, collaboration, role of mother, role of father, role of siblings, role of social environment, language development, academic development, social development, impact of parent education
**Guidance**		
Rights Education Accessibility	(6) Rights, (11) facilitating decisions about the child, (5) educational opportunities, (10) psycho-social support resources, (36) accessibility to specialist	Education, health, hearing technology, decisions, educational rights, supporting family, meeting with other families, psychological understanding, social support, referral, access to specialist
**Intervention**		
Interaction Routines Materials Listening skills Play Behavior management Role of specialist	(13) establishing joint attention, (14) making eye contact, (15) taking turns, (16) quality interaction environment, (20) enriching expressions, (21) using repetitions, (19) using routines, (17) material selection, (12) developing listening skills, (28) daily device control, (18) play selection, (23) behavior management, (38) attitude of the specialist, (39) sensitivity to cultural characteristics, (22) home recommendations	Joint attention, eye contact, turn taking, information, observation, modeling, routines, toys, storybooks, real objects, pausing, asking questions, establishing eye contact, home plays, appropriate play selection, approach to the child, behavior management, desired behavior, undesired behavior, attitude of the specialist
**Assessment**		
Child assessment Parent assessment	(26) Supporting developmental areas, (27) assessing general development, (34) assessing the child, (29) determining the educational needs, (35) assessing the family, (37) motivating the family, (38) home visits, (32) observing the interaction of the family	Assessment of language development, monitoring development, assessment of development areas, parent development, supporting the family, home visit, feedback, observing the interaction, monitoring

**Table 4:** Joint-display of the findings of information dimension.

Sub-dimensions	Quantitative findings (RQ-1)		Extract of qualitative findings (RQ-2)	Metainference codes (RQ-3)
	Item content	Yes (%)		
Nature of hearing	Effects of hearing loss Communication mode Hearing technology	92.8 90.2 96.2	Parents stated that they were referred to cochlear implantation without adequate information and that they were not informed about hearing loss.	Discordance Expansion
Parent education process	Sharing education content Sharing education purpose	94.7 94.7	Parents stated that not enough information was shared about the purpose and content of the education*.*	
Role of family/social environment	Parental attitudes Family role in language development The child’s place in the family The role of social environment in language development Approach to social reactions to hearing loss	90.2 96.2 74.7 87 70.9	Parents stated that they were not informed about the role of the family and social environment and that they learnt many issues by experiencing them themselves. Only one parent stated that the close environment was included in the parent education process.	Discordance Expansion Confirmation
Realistic expectation	Setting realistic expectations	87.6	Parents stated that realistic expectations were established in parent education process.	Confirmation

*Note.* RQ = research question.

The four dimensions (information, guidance, intervention, and assessment) presented in Table 3 serve as a framework for quantitative and qualitative analyses. The researchers transcribed the recorded semi-structured interviews and ensured their accuracy. The qualitative data were coded, and consensus was reached on the coding process. A total of 57 codes were identified and categorized into four main groups: 15 codes for information, 11 for guidance, 22 for intervention, and 9 for assessment.

Joint-display tables (Tables 4–7) were used to convert analyses into integrated findings. In these tables, quantitative and qualitative findings were presented side by side, followed by metainference codes that synthesized these findings (Corrigan & Onwuegbuzie, 2020; Fetters & Guetterman, 2021). For example, in Table 4, under the quantitative findings’ column (RQ-1), 92.5% of parents answered “Yes” to the survey item on “hearing technologies”. However, in the qualitative findings column (RQ-2), some parents reported, “We were referred to cochlear implantation without adequate information”, which does not fully align with the quantitative findings. In RQ-3, the terms “discordance” (for the above example), “expansion”, or “confirmation” were used as metainference codes to indicate the consistency between the findings. Metainferences are descriptions, conclusions, or insights derived from integrating quantitative and qualitative data in mixed methods research (Younas et al., 2023). Therefore, metainferences reflecting the content of the codes are provided as text below each table.

### Legitimacy

In mixed-methods research, the concept of “legitimacy” represents the counterpart of validity and reliability in quantitative and qualitative research (Leko et al., 2023). This study follows the criteria suggested by Leko, which depends on Johnson and Christensen (2020). Key criteria include multiple validities, commensurability approximation, integration, and weakness minimization. To ensure *multiple validities*, the credibility criteria for the qualitative dimension (Glesne, 2016) and the principles of survey development for the quantitative dimension (Cohen et al., 2021; DeVellis & Thorpe, 2021) were followed. The careful attention given to utilizing both quantitative and qualitative findings in line with the research objectives demonstrates a focus on *commensurability approximation.* The *integration* criterion was applied throughout the study, from research design to formulating research questions, selecting participants, and presenting and discussing findings, with quantitative and qualitative results integrated and reported together. For *weakness minimization*, the power of sample representation was strengthened through quantitative data, and qualitative findings were used to explain potential causes. This approach ensured that the strengths of both quantitative and qualitative methods were leveraged.

## Findings

To assess the quality of FCEE provided to parents of DHH children aged 0–3, both quantitative and qualitative findings were analyzed and reported across four key dimensions: information, guidance, intervention, and assessment. Within each dimension, responses to all research questions were presented comparatively to provide a comprehensive understanding of the findings.

### Information

The joint display of the quantitative and qualitative findings for the information dimension is shown in Table 4.

As shown in Table 4, the highest level of information (96.2%) was related to hearing technologies and the family’s role in language development, while the lowest (70.9%) pertained to addressing social responses to hearing loss. However, data from the parent interviews revealed significant discrepancies compared to the survey findings. Except for one participant, all parents reported that information was either not provided or was insufficient. For instance, Participant M1 stated that they received no guidance on how to communicate with their child and instead attempted to resolve the issue by observing the teacher: “I was watching [child’s name]’s lesson, and during that time, I believe families should be informed.” Participants who felt inadequately informed reported turning to alternative sources such as the internet, social media, and other parents of DHH children. The only participant who reported a positive experience, M5, noted that extended family and close acquaintances were included in the educational process: “We took his grandmother with us, and she joined. We have a very close friend, his spiritual grandmother, and they even included her in the lesson… anyone around the child was welcomed.” Participant M2 reflected on the overall quality of the FCEE by stating, “You can’t really assess whether the training is good or bad. Honestly, we were very lucky in Türkiye.” The metainference of this dimension is that the information provided to the parents is inadequate concerning the nature of hearing, the parent education process, and the role of the family and social environment. However, it is sufficient for helping parents establish realistic expectations.

### Guidance

The joint display of the findings for the guidance dimension is shown in Table 5.

**Table 5:** Joint-display of the findings of guidance dimension.

Sub-dimensions	Quantitative findings (RQ-1)		Extract of qualitative findings (RQ-2)	Metainference codes (RQ-3)
	Item content	Yes (%)		
Rights	Information on rights	73.5	Parents stated that they did not receive adequate guidance on their rights and that they learnt their rights through experience.	Discordance Expansion
Education	Facilitating decisions about the child Education opportunities	81.7 87.7	Parents stated that they did not receive sufficient guidance about decisions and educational opportunities.	
Accessibility	Psycho-social support resources Accessibility to specialist	72.9 91.3	Parents could not receive psycho-social support, and some parents had to change provinces to access specialists.	

*Note.* RQ = research question.

Table 5 shows an apparent discrepancy between the quantitative and qualitative findings across all subdimensions of the guidance. According to the survey results, parents reported receiving the least guidance regarding psycho-social support resources (72.9%) and the most regarding accessibility to specialists (91.3%). However, qualitative interviews raise concerns about the quality of the guidance provided. Parents reported insufficient support in areas such as legal rights, educational opportunities, decision-making, and psycho-social support. Even in accessing specialists, some parents faced notable challenges. For example, Participant M6 described a demanding travel experience: “I was rushing to get that training… there was not even a high-speed train back then. Three and a half hours with my child on my lap!” While specialists are expected to assist parents in decision-making, Participant F1 described being left to make difficult decisions alone: “Some decisions about my child are really tough. Some of the things recommended by the institution and our teachers are not easy to implement.” Likewise, Participant M4, who reported inadequate guidance across all areas, emphasized: “We actually learned about our legal rights only after facing problems ourselves.” The metainference of findings related to guidance indicates that, despite some guidance being provided in the subdimensions of rights, education, and accessibility, parents generally did not consider it adequate.

### Intervention

The joint display of the findings for the intervention dimension is shown in Table 6.

**Table 6:** Joint-display of the findings of intervention dimension.

Sub-dimensions	Quantitative findings (RQ-1)		Extract of qualitative findings (RQ-2)	Metainference codes (RQ-3)
	Item Content	Yes (%)		
Interaction	Joint attention Making eye contact Turn taking Quality interaction environment Enriching expressions Utilizing repetitions	90.5 90.8 91.6 86.8 90.9 94.3	Parent interviews showed that there were different experiences in all of the intervention sub-dimensions. In each sub-dimension, there are experiences that are compatible with the quantitative findings as well as incompatible experiences. For example, while one parent received adequate support for play selection, another parent stated that they did not receive support for it.	Discordance Expansion Confirmation
Routines	Utilizing routines	91.6		
Material	Selection of appropriate materials	89.7		
Listening skills	Developing listening skills Daily device check	93.8 85.6		
Play	Play selection	90.1		
Behavior	Behavior management	90.5		
Role of specialist	Specialist’s attitudes Sensitivity to cultural characteristics Home recommendations	97 84.7 95.1		

*Note.* RQ = research question.

Table 6 shows that participants reported interventions within FCEE were implemented across subdimensions at rates ranging from 84.7% to 95.1%. However, interviews with parents revealed varying experiences across these subdimensions. While some parents benefited from appropriate interventions in certain areas, others reported receiving insufficient support. For instance, Participant M5 described the interactive nature of the sessions, stating, “They involve you in the games, which gives you the opportunity to practice. So, you are not just passively observing.” In contrast, Participant M6 expressed dissatisfaction, stating, “...there were no game suggestions or anything like that.”

Another striking qualitative finding within this dimension related to the attitude of specialists. One participant reflected critically on a specialist’s demeanor during the period of their child’s diagnosis by stating:

Yes, they may see many children like this. However, how many families are like us, experiencing their first child, their first excitement, only to feel like boiling water has been poured over them? Then you are faced with someone who responds indifferently and says, “Just accept it.” You are crying, and they tell you, “Go ahead and cry, it will make you feel better.” However, you are expected to accept it, yes, your child is deaf (M5).

Although the highest percentage of “Yes” responses in the survey was related to this dimension, the interviews revealed that parents did not benefit equally from the intervention services. The metainference regarding the findings on the intervention dimension indicated that, while almost all subdimensions (interaction, routines, materials, listening skills, play, behavior management, expert role) were addressed in the training, parents did not experience a functional or applicable training process in several subdimensions, such as play, listening skills, and behavior management.

### Assessment

The joint display of the findings for the assessment dimension is shown in Table 7.

**Table 7:** Joint-display of the findings of assessment dimension.

Sub-dimensions	Quantitative findings (RQ-1)		Extract of qualitative findings (RQ-2)	Metainference codes (RQ-3)
	Item content	Yes (%)		
Child assessment	Supporting development areas Assessing the general development Assessment of the child	89.4 92 92	The parents stated that there was no systematic assessment of the children, and that the assessment was made in short after the sessions.	Discordance Expansion Confirmation
Parent assessment	Determining the educational needs of the family Observing the interaction of family Home visits Assessing the family Motivating the family	82.5 83.8 21.9 86.8 86.4	The parents stated that while they were assessed in the form of teacher feedback after the sessions, home visits and parent–child interaction were not observed.	Discordance Expansion

*Note.* RQ = research question.

As shown in Table 7, the quantitative findings showed that the assessment dimension of FCEE focused primarily on child assessments (92%), while very few home visits (21.9%) were conducted. Other survey items also revealed a relatively high percentage of “Yes” responses. For example, 89.4% of parents agreed with item 26 “The aspects of my child that needed to be developed were shared”, and 92% agreed with item 27 “Information was given about the general development of my child”. However, the interview data indicated no systematic assessment was conducted for either children or parents. The assessment practices typically consisted of brief feedback provided by teachers to parents after lessons. Participant M8’s statement, “We only receive a daily assessment of the lessons from our teacher, but aside from that, we have not received any institutional assessments.” confirmed this finding. A parent with different experiences reported that the assessment was documented and more detailed.

Each child has a notebook kept by their teacher, as you know. It records what the child has and has not done throughout the week. The teacher reviews it and checks: “Where is [child’s name]? What have they done, and what haven’t they done? What is missing?’ Then, they might say, ‘Let us add this here” (M4).

Most parents reported issues with family assessment, increasing family motivation, and home visits. The metainference from these findings is that, although a systematic assessment process exists for the child, no comparable assessment process is in place for the parents.

## Discussion

This study explored the extent to which FCEE services were provided to parents of children aged 0–3 in Türkiye, examined parents’ perceptions of these services, and presented metainferences based on the findings. The most striking aspect of the findings is the discrepancy between the quantitative and qualitative results. While the quantitative data indicate that the services provided are primarily knowledge-based, the qualitative findings raise concerns about the quality of those services. Several factors may explain this inconsistency. First, surveys may be subject to response bias since they rely on self-reported data (Cook, 2014). In this study, 96% of the participants were parents of children attending SERCs. As noted in the introduction, SERCs are private institutions that provide services under the MoNE. Although the informed consent form assures participants that their data will be used for research purposes and remain anonymous, parents may feel compelled to protect the institution that educates their children, fearing that negative evaluations could affect the quality of services received.

The second and more likely possibility relates to the structure of the FCEE-DHH Survey. While the survey identifies whether a particular characteristic is present, it cannot capture the processes underlying that characteristic. For instance, parents generally responded “Yes” to the statement, “I was given information that made it easier for me to make decisions about my child” (Survey item 11). However, the survey does not reveal the nature of the information provided, i.e., who provided it, in what context, and with what content, using the survey question. To address these gaps, semi-structured interviews were employed. Given the critical perspective they offer and the complementary and expansive function of mixed methods research (Leko et al., 2023), qualitative data were given priority in the interpretation of findings. The Likert-type design of the survey may yield more efficient results in determining the intensity of early intervention in future studies.

The findings indicate that the way parents participate in FCEE programs varies by institution. This variation includes differences in the duration, frequency, and assessment methods of the training. Since there is currently no widely adopted parent education program, it can be concluded that FCEE practices in Türkiye may lack standardization. Deficiencies were found in the information dimension, with parents turning to internet resources and other parents of DHH children to fill these gaps. In the guidance dimension, parents faced challenges in being directed to psycho-social support resources, and their psycho-social processes were often overlooked. In the intervention dimension, parents’ participation in training was insufficient, and the training was predominantly child-centered. Additionally, parents were not provided with a systematic assessment of the process of either their children or themselves during the FCEE practices. These assessment gaps may lead to unrealistic expectations about their children’s development. Understanding the reasons for these shortcomings based on parents’ needs and experiences with FCEE may inform the development of targeted interventions and policies.

### The current state of FCEE

Parents reported no significant difficulties accessing services during the diagnosis and amplification stages of early intervention. International guidelines recommend that FCEE services begin promptly after diagnosis (JCIH, 2019; Walker et al., 2022). Countries like the United States, Australia, and Norway have successfully implemented newborn hearing screening programs. However, such programs are not consistently available across all European countries, with Greece, for instance, still facing challenges in this area (Athanasopoulos et al., 2024). The consistent implementation of these programs is seen as a prerequisite for ensuring timely access to early education during the critical early years (Holzinger et al., 2011). During this process, parents make important decisions regarding communication approaches, implants, and educational opportunities for their children (Athalye et al., 2015; Bruder & Dunst, 2005; Hopkins & Puhlman, 2025), which creates a considerable need for information, particularly for those encountering hearing loss for the first time after their child’s birth.

For parents to make informed decisions for their children, they must have access to adequate information (Hopkins & Puhlman, 2025; JCIH, 2019). Survey results in Türkiye indicated that parents received information on topics such as the nature of hearing, the parent education process, the role of the social environment, and setting realistic expectations. However, interview data suggested that this information was insufficient. This lack of adequate guidance makes it more difficult for parents to make informed decisions about their children (Jackson et al., 2008) and diminishes the overall effectiveness of early intervention (Bruder & Dunst, 2005; van der Spuy & Pottas, 2008). When parents feel competent in their decision-making and their needs are met, they tend to feel more empowered (Jackson et al., 2008; Sass-Lehrer et al., 2016).

Qualitative findings also indicated that gaps in the provision of information influenced parents’ attitudes toward and expectations of FCEE. Moreover, when adequate information was not provided, parents often sought out knowledge and resources on their own. Fitzpatrick et al. (2025) have reported that this situation prolongs the time required for diagnosis, device fitting, and access to education. Similarly, a study conducted in the U.S. revealed that parents who faced challenges in obtaining information also struggled to access resources from the point of diagnosis onward (Vukkadala et al., 2019). In the language development of DHH children, the quality of interactions within their linguistic environment plays a decisive role (Vygotsky, 1978). A parent’s ability to structure and enrich this environment is closely linked to the fulfillment of their informational needs (Bruder & Dunst, 2005). Similarly, the broader social environment, including the extended family, significantly influences the child’s language development (Bronfenbrenner & Evans, 2000; Costa et al., 2019; Ingber & Dromi, 2010). In Türkiye, however, the resources currently provided appear to fall short in informing and guiding parents regarding the role of the social environment.

Both the survey results and interview findings indicated that parents were not sufficiently guided toward psycho-social support resources. Since parents often lacked prior experience with hearing loss and its implications (Dirks et al., 2019), they might face significant emotional challenges from the moment they receive their child’s diagnosis (Luterman, 2017). To help parents adjust to this new reality and manage the emotional impact, it is essential to provide accurate information and refer them to appropriate psycho-social support resources (Aksoy et al., 2023; Moeller et al., 2013; Moeller et al., 2024b). The study revealed that although parents may have access to psychologists at SERCs, they often did not receive adequate psychological support. Available psycho-social support resources include psychologists working in SERCs (İçyüz & Doğan, 2023), psychological counselors in Guidance and Research Centers (GRCs), and peer support from other parents of DHH children (Kılıç, 2020; Zaidman-Zait & Most, 2005). Properly informing and directing parents to these resources is critical to supporting them in the decision-making process regarding their children.

In Türkiye, the resources provided to parents were primarily focused on information, referrals, and financial assistance in areas such as diagnosis, hearing aid amplification, cochlear implantation, and education, while psychological and emotional support needs were often overlooked. It is emphasized that the psycho-social difficulties experienced by families must be addressed at the individual (micro), social (meso), and political (macro) levels (Kecman, 2022). A recent study conducted in China also found that while parents received substantial financial support during early intervention, very little emphasis was placed on psychological support (Xiong & Li, 2025). SERCs are widespread and accessible to most families across Türkiye (Atmaca & Uzuner, 2020), which makes them a viable setting for delivering adequate psychological support through on-site psychologists. Additionally, specialists can refer families to GRCs for further counseling services. Educators can also play a role in facilitating psycho-social support by organizing playgroups within early childhood institutions, where parents of DHH children aged 0–3 can connect with one another (Turan, 2021). Since it is expected that well-supported parents will make more informed decisions regarding their children (Bruder & Dunst, 2005), empowering them as active decision-makers is critical (Hopkins & Puhlman, 2025).

There are significant inconsistencies in how interventions aimed at helping parents support their children’s language development are implemented across different cities, specialists, and intervention centers. FCEE should include routines, activities, and play that actively promote language development (Clark, 2007; Cole & Flexer, 2020; Holzinger et al., 2022). However, in Türkiye, such practices are not uniformly delivered to all parents. While some parents reported that their FCEE experiences included language-enhancing strategies, many others either lacked these practices or found their implementation insufficient.

Parental experiences in Türkiye often diverge from international standards (Moeller et al., 2013). One significant discrepancy is that FCEE services tend to be primarily child-centered, while global best practices emphasize a family-centered approach that supports the entire family (JCIH, 2019). A key finding of this study was that parents were frequently asked to wait outside the classroom during their child’s sessions, rather than being actively included in the educational process. In a well-implemented FCEE program, specialists should explain the purpose of specific techniques used to support spoken language and listening development, helping parents understand how these activities contribute to specific developmental goals (Brown & Nott, 2005; Moodie et al., 2024). Beyond explanations, parents should be actively engaged through demonstrations and practice, which enable them to gain the skills and confidence needed to support their child’s learning at home (Bruder & Dunst, 2005; Dunst & Espe-Sherwindt, 2017; Turan, 2014). However, this study found that even when parents were present during sessions, their involvement was largely passive, limited to observation. This lack of active participation hinders parents’ ability to reinforce language development in everyday settings outside the formal intervention environment (Holzinger et al., 2022). When specialists actively involve parents in sessions alongside their children, parents are better positioned to understand the pace and nature of their child’s progress (Turan, 2014), thereby becoming more effective partners in the developmental process.

The findings suggested that parents were not being adequately assessed regarding both their children’s developmental progress and their own progress in acquiring the skills needed to support language development. Specialists are expected to provide parents with regular updates on their child’s progress, offer constructive feedback, and guide them in becoming effective observers of their child’s development (Dunst & Espe-Sherwindt, 2017). Active parental involvement in the intervention process not only enhances motivation but also plays a critical role in supporting the child’s developmental outcomes (Zaidman-Zait et al., 2018). When parents feel confident in their knowledge and skills, their sense of competence significantly enhances their interactions with their children (Sass-Lehrer et al., 2016). However, the lack of systematic assessment makes it challenging to track parents’ progress or provide meaningful feedback. Therefore, there is an urgent need to develop structured assessment tools to monitor parental development throughout the FCEE practices and ensure consistent support.

Parents also reported difficulties in managing their children’s behavior. Research conducted in both Türkiye (Sevinç & Şenkal, 2021) and Iran (Eyalati et al., 2013) indicates that addressing behavioral issues can be particularly challenging for parents. Active parental involvement in the intervention process allows them to observe how specialists address behavior and discipline, enabling them to learn by example. It is also essential for specialists to provide concrete assessments of the child’s behavior to help parents adjust their expectations to more realistic levels. At the start of early FCEE, parents may develop either high expectations for rapid progress or concerns that no progress will be made at all, both of which can lead to disengagement from the process (Doğan, 2015; Luterman, 2017). Unrealistic expectations may cause families to perceive themselves, early intervention, or their children as “failures” (Mauldin, 2019).

Quantitative findings indicated that home visits were rarely conducted. A study from South Africa, like findings in Türkiye, identified home visits as a crucial factor in ensuring parental involvement in early intervention (Maluleke et al., 2021). Home visits allow specialists to observe parent–child interactions in both educational and other environments. These visits can support comprehensive assessments and help establish realistic expectations.

### How can international standards be achieved in FCEE?

The success of FCEE applications depends on establishing a bridge between policies and practices. The most effective way to do this is through practices sensitive to family needs (Petrocchi-Bartal et al., 2025), individual and cultural characteristics (Odom, 2016; Vygotsky, 1978). In Türkiye, newborns undergo hearing screening through NNHSP, which enables the early diagnosis of DHH children. Once diagnosed, children are provided with hearing devices supported by the government, which partially covers hearing aids and fully covers cochlear implants. Following amplification, FCEE is primarily delivered through SERCs. Parents are entitled to two hours of free training per week at SERCs (MoNE, 2018), and 96% of participants in this study reported receiving such services. As a result, nearly all parents receive education four times a month. While diagnosis, amplification, and educational resources are provided at no cost, the primary concerns regarding FCEE lie in the quality of the educational resources. This highlights the urgent need for clearly defined parent education policies, evidence-based and consensus-driven training programs, and the professional development of specialists. To address these needs, a collaborative protocol could be established between the MoNE, the Ministry of Health, and the Ministry of Family and Social Services. Such a tripartite agreement would provide a comprehensive roadmap for both parents and specialists, beginning from the point of diagnosis. With broad stakeholder involvement, this protocol could establish a nationally agreed-upon, adaptable, and internationally aligned framework for delivering high-quality FCEE services.

While parents did not directly comment on the qualifications of specialists, some reported relocating to another province to access better educational resources for their children, and 64.6% received support from more than one specialist. In addition, at least 15.8% of specialists are not hearing-speech specialists. These findings indicate that the qualifications of specialists are not sufficient enough. For FCEE to be implemented in Türkiye in alignment with international standards, the qualifications of specialists must be enhanced. Regular training and certification programs can be organized to enhance the qualifications of specialists, especially for those working in SERCs. These institutions can be strengthened in terms of the psychosocial support services they offer to parents. Parents’ relocation for education also creates additional financial burdens (Xiong & Li, 2025), which could be alleviated through telepractice applications that provide FCEE services without geographical barriers (Behl et al., 2017; McCarthy et al., 2020).

International guidelines emphasize the importance of interdisciplinary collaboration among specialists involved in FCEE (Szarkowski et al., 2024), and there is a clear need to strengthen such cooperation within Türkiye as well. Moreover, increasing parental awareness of FCEE services is essential for enhancing service quality. Informed parents are more likely to advocate for high-quality education and to demand consistent, evidence-based support for their children.

The findings of this study highlight the clear need for the development of systematic parent education programs tailored to parents of DHH children aged 0–3. These programs should be designed through interdisciplinary collaboration involving audiology, special education, deaf education, speech and language therapy, early childhood education, and psychology. Strengthening cooperation among the education, health, social service providers, and universities is crucial to ensure the integrated delivery of early intervention services.

Finally, comparative studies with countries that have achieved high standards in FCEE could help inform the development of quality benchmarks for Türkiye and serve as a model for other non-Western contexts.

### Limitations

The most significant limitation of the study appears to be the disparity in the number of participants between the quantitative (*n* = 268) and qualitative (*n* = 10) phases. Although the main purpose of explanatory sequential mixed methods research is to explain, expand, and deepen quantitative findings through qualitative data (Leko et al., 2023), having more participants in the qualitative phase could have provided a richer and more nuanced understanding of the FCEE picture in Türkiye.

The participants in the qualitative phase of the study were selected from the quantitative phase based on certain parameters. These parameters did not include the characteristics of the service providers. Therefore, it was not possible to determine from the quantitative data whether the discrepancies between the two types of findings were related to the characteristics of the service providers. Comments on how specialist qualifications affect the quality of FCEE were limited to the statements of a few participants.

The study was conducted with parents living in 35 different cities in Turkey, 96% of whom received services from SERCs, which may enhance the generalizability of the findings. However, the large number of SERCs and their commercial nature made comparisons between them infeasible. Future studies could examine the potential effects of SERCs by limiting the number of cities and SERCs, increasing the number of participants, and anonymizing the centers.

The participants included parents who are currently undergoing the FCEE process, as well as those who have recently completed the process, and have relatively recent experiences. However, because the time elapsed between the completion of the FCEE process and data collection was not specified, the timeliness of the perspectives of parents who had completed the process remains uncertain. This situation may lead to a risk of memory bias. Nonetheless, including these parents in both research phases was considered meaningful for the integrity of the findings.

Finally, the participants represent a highly heterogeneous population (see Table 1), in that, they differ in terms of educational status, closeness to their child, hearing technology, communication approach, additional disabilities, educational institution, frequency of education, and type of early intervention specialist. For example, 80% of the participants were mothers, which may have limited the representation of fathers’ experiences and perspectives regarding AMEE (Acar et al., 2020; Szarkowski & Dirks, 2021). Except for some views reported in the qualitative phase, this study did not directly address the impact of the above-mentioned characteristics on FCEE. Readers are expected to consider these limitations when interpreting the findings.

## Supplementary Material

APPENDIX_A_FCEE_SURVEY_enaf081

APPENDIX_B_INTERVIEW_QUESTIONS_enaf081
