# Supplementary material for: Where are we in family-centered intervention? Parental experiences of DHH children
Source: J Deaf Stud Deaf Educ. 2025 Dec 31;31(3):495–509. doi: 10.1093/jdsade/enaf081 (PMC13343197; doi:10.1093/jdsade/enaf081)
Supplement: APPENDIX_A_FCEE_SURVEY_enaf081 [file appendix_a_fcee_survey_enaf081.docx]

**Appendix A**

| **SECTION 1**  **DEMOGRAPHIC INFORMATION** | | | |
| --- | --- | --- | --- |
| *This section includes questions about you and your child. Please answer each question carefully.* | | | |
| 1. How old is your child?   O 1  O 2  O 3 | O 4  O 5  O 6 | | |
| 1. What is the gender of your child?   O Female  O Male | | | |
| 1. How old was your child when he/she was diagnosed with hearing loss?   _______ month(s) | | | |
| 1. Which hearing technologies does your child use?   O Does not use hearing aids or implants.  O Wears a hearing aid (in one or both ears).  O Uses a cochlear implant (in one or both ears).  O Wears a hearing aid in one ear and a cochlear implant in the other.  O Uses a brain stem implant. | | | |
| 1. Which communication approach do you want your child to use?   O Spoken language  O Sign language  O Both of them | | | |
| 1. Which communication approach does your child use the most?   O Spoken language  O Sign language  O Both of them | | | |
| 1. Does your child have a diagnosed disability other than hearing loss?   O No  O Yes [*please* *specify* ___________________________] | | | |
| 1. Is there anyone in your family with hearing loss other than your child? *[You may select more than one option].*   O Mother  O Father  O Brother  O Other | | | |
| 1. What is your relationship with the child?   O I am his/her mother.  O I am his/her father.  O Other [*please specify* ___________________________] | | | |
| 1. What is your educational level?   O No education/literate  O Primary School  O Secondary School/Primary Education  O High School  O University  O Postgraduate | | | |
| 1. Which city do you live in?   ____________________________ | | | |
| 1. In which city did you participate education when your child was 0-3 years old?   ____________________________ | | | |
| 1. How long have you participated education since the diagnosis?   _______ month(s) | | | |
| 1. In which institution did you participate education in the 0-3 age group? *[You may select more than one option].*   O Special education and rehabilitation center  O Preschool for hearing children  O Daycare center for hearing children  O Special education kindergarten  O Appied Research Center for Hearing Impaired Children (İÇEM, Turkish abbreviation)  O Hospital  O Private practice (clinic)  O Other [*please specify* ___________________________] | | | |
| 1. From which specialist did you have education in the 0-3 age period? *[You may select more than one option].*   O Teacher for the hearing impaired  O Special education teacher  O General education teacher  O Preschool teacher  O Child development specialist  O Audiologist  O Speech and language therapist  O Psychologist  O Other [*please specify* ___________________________] | | | |
| 1. How often did you participate education when your child was 0-3 years old?   O 1 per month  O 2 per month  O 4 per month  O 8 per month  O Other [*please specify* ___________________] | | | |
|  | | | |
| **SECTION 2**  **FAMILY-CENTERED EARLY EDUCATION SURVEY-DHH** | | | |
| *This section includes statements about the quality of the education you and your child participated when your child was 0-3 years old. Please check* **Yes** *if the statement reflects the content of the family education you participated in, and* **No** *if it does not.* | | | |
| **Statements** | | **Yes** | **No** |
| 1. I was given information about how hearing loss affects my child's development. | |  |  |
| 1. Information was given on how people with hearing loss communicate (spoken language, sign language etc.). | |  |  |
| 1. Information on the use of hearing aids or implants was provided. | |  |  |
| 1. The role of the family in my child's language development was explained. | |  |  |
| 1. Pre-school education opportunities were explained. | |  |  |
| 1. We were informed about our rights. | |  |  |
| 1. I was told how to approach people's reactions to my child's hearing loss. | |  |  |
| 1. The role of the social environment in my child's language development was explained (relatives, close environment, friends, peers, etc.). | |  |  |
| 1. I was informed about what I can do in case of negative interactions between family members (e.g. sibling jealousy, excessive interest in one of the children) | |  |  |
| 1. I was given information about the psycho-social support I can get when I need it (e.g. experts, other families, non-governmental organizations) | |  |  |
| 1. I was given information that made it easier for me to make decisions about my child. | |  |  |
| 1. I was informed on what I will do to improve my child's listening skills. | |  |  |
| 1. I was informed on how to establish joint attention in interaction with my child. | |  |  |
| 1. I was told how to make eye contact when interacting with my child. | |  |  |
| 1. I was told that we should take turns in interacting with my child. | |  |  |
| 1. Suggestions were made for a quality interaction environment. | |  |  |
| 1. The selection of materials (toys, storybooks, etc.) appropriate to my child's level was explained. | |  |  |
| 1. I was told how to identify appropriate plays for my child's level. | |  |  |
| 1. I was informed on how to use daily life opportunities to support my child's language development. | |  |  |
| 1. I was told how to enrich my child's expressions to improve his/her language skills. | |  |  |
| 1. I was told how to make my child repeat his/her statements to improve his/her oral language skills. | |  |  |
| 1. Suggestions were given on what we could do with my child at home. | |  |  |
| 1. I was told how to intervene in my child's undesired behaviors. | |  |  |
| 1. I was informed about the parental attitude I should exhibit towards my child. | |  |  |
| 1. The level of development my child could reach after the education was shared with me. | |  |  |
| 1. The aspects of my child that needed to be developed were shared. | |  |  |
| 1. Information was given about the general development of my child (e.g. physical, cognitive, social, emotional, motor development). | |  |  |
| 1. My child's daily device checks were done regularly. | |  |  |
| 1. At the beginning of the education, we identified our education needs as a family. | |  |  |
| 1. At the beginning of the education, the objectives of the education were shared. | |  |  |
| 1. Information about the content of the education was given at the beginning of the education. | |  |  |
| 1. What I did with my child during the education was observed. | |  |  |
| 1. Visits were made to our home by the specialist who provided the education. | |  |  |
| 1. Information was shared regularly about how my child was developing. | |  |  |
| 1. I was regularly informed about my educational progress as a parent. | |  |  |
| 1. The specialist was available when I needed him/her. | |  |  |
| 1. It emphasized what I do well as a parent | |  |  |
| 1. The attitude of the specialist was supportive. | |  |  |
| 1. Our family characteristics were considered during family education. | |  |  |
| 1. What else would you like to add about the education you and your child participated in?   ... | |  |  |
