# Supplementary material for: Where are we in family-centered intervention? Parental experiences of DHH children
Source: J Deaf Stud Deaf Educ. 2025 Dec 31;31(3):495–509. doi: 10.1093/jdsade/enaf081 (PMC13343197; doi:10.1093/jdsade/enaf081)
Supplement: APPENDIX_B_INTERVIEW_QUESTIONS_enaf081 [file appendix_b_interview_questions_enaf081.docx]

**Appendix B**

**Semi-Structured Interview Questions**

(Please review the interview questions using the framework provided in Table 3 and the accompanying explanations in the article)

1. Can you briefly introduce yourself and your child?
2. What were your opinions about family education before you participated in education?
3. How did you begin family education?
   1. How did you decide?
   2. When? Where? With which specialist? How often?

4) Can you tell us about the family education process you participated in?

a. Planning phase

b. Implementation phase

c. Evaluation phase

d. Monitoring phase

1. a) What would you say about your role in family education?

b) What would you say about the role of other stakeholders (other family members, [specialist](https://tureng.com/tr/turkce-ingilizce/specialist)s , educational institutions, relevant government agencies)?

1. How do you think family education influences your decisions about your child?
   1. Communication approach
   2. Hearing technologies
   3. Education
   4. Supporting language development
2. What were the strengths and weaknesses of the family education you participated in?
3. What are your suggestions for improving the family education offered (close environment, relevant government agencies, families, [specialist](https://tureng.com/tr/turkce-ingilizce/specialist)s, educational institutions)?
4. How has the education changed your and your child's life?
5. What do you think a good family education should looks like?
6. How would you evaluate existing family education practices in this context?
7. Is there anything you would like to add?
8. May I see you again?
